# OLDER AUSTRALIAN WOMEN’S HUMAN CAPITAL: PERCEPTIONS OF SKILL LEVELS AND PARTICIPATION IN LEARNING ACTIVITIES

**DOI:** 10.1093/geroni/igz038.011

**Published:** 2019-11-08

**Authors:** Philip Taylor

**Affiliations:** Federation University Australia, Melbourne, Victoria, Australia

## Abstract

While business cases for older workers’ employment stress their value it is well-known that they participate less in training activities than younger workers. Women are at particular risk of not accessing training opportunities. Drawing on a survey of 2500 women aged over 50 we report a fine-grained analysis of the types of training women were undertaking and the factors associated with participation in training. The analysis indicates that training is infrequently undertaken in preparation for a new job and large majorities of women see no need to and are not interested in retraining. This is observed across occupational groups, but more commonly among those with low educational levels. A lack of employer support is much less commonly reported as a barrier to older women’s participating in training. The findings suggest that it is primarily at women themselves that efforts aimed at promoting human capital development need to be directed.

